# The effect of reproductive hormones on women’s daily smoking across the menstrual cycle

**DOI:** 10.1186/s13293-021-00384-1

**Published:** 2021-06-10

**Authors:** Ashley R. Ethier, Ty L. McKinney, Laurie Sykes Tottenham, Jennifer L. Gordon

**Affiliations:** 1 Department of Psychology, University of Regina, Regina, Saskatchewan Canada; 2 Department of Psychology, College of Social and Behavioral Science, University of Utah, Salt Lake City, Utah USA

**Keywords:** Reproductive hormones, Estrogen, Estradiol, Progesterone, Estrone-3-glucuronide (E1G), Pregnanediol glucuronide (PdG), Urinary metabolites, Menstrual cycle, Smoking behavior

## Abstract

**Background:**

Women attempt to quit smoking less often than men and are less likely to maintain abstinence. Reproductive hormones have been postulated as a reason for this sex difference, though this remains to be clarified. Research suggests that estradiol and progesterone may influence nicotine addiction, though various methodologies have led to inconsistent findings. The current study aimed to directly examine the effect of reproductive hormones on women’s smoking behavior.

**Methods:**

Over the course of one menstrual cycle, twenty-one female smokers recorded the number of cigarettes smoked in a day, as well as their perceived need for and enjoyment of cigarettes smoked. Additionally, they provided 12 urine samples for the measurement of the urinary metabolites of estradiol (estrone-3-glucuronide, E1G) and progesterone (pregnanediol glucuronide, PdG). Multilevel modeling was used to examine the effects of hormone levels as well as hormone change on smoking outcomes.

**Results:**

When PdG levels were low, they were inversely associated with daily cigarettes smoked. Furthermore, E1G level was negatively associated with both self-reported need for and enjoyment of cigarettes smoked but not the number of cigarettes smoked. Examining the effect of hormonal change on smoking outcomes revealed a significant interaction between change in PdG and E1G on number of cigarettes smoked such that only a simultaneous drop or increase in both hormones was associated with a greater number of cigarettes. Hormonal change effects on need for and enjoyment of cigarettes were not significant.

**Conclusions:**

The present study suggests that (1) elevated progesterone levels lessen the propensity to smoke in women, (2) estrogen levels influence women’s subjective experience of smoking, and (3) simultaneous drops or increases in these hormones are associated with increased smoking.

## Background

Smoking tobacco is extremely detrimental to one’s health, its link with nearly every existing chronic health condition well-documented [1]. In spite of this, the World Health Organization (WHO) estimates that 20% of the world’s population over the age of 14 currently smokes [2]. As a result, tobacco use is attributable to eight million deaths per year and smoking cessation is considered the most effective way to reduce worldwide morbidity and mortality [3]. While quitting is a common desire among those who smoke, only one third of individuals actually attempt to quit; of those who do attempt to quit, 75–80% fail to achieve 6 months of abstinence [4]. In fact, research suggests that the average smoker attempts to quit at least 30 times before successfully remaining abstinent for 1 year [5].

The tendency for smoking cessation failure largely relates to the reinforcing properties of nicotine, which is the addictive element of cigarettes [6]. Like other addictive substances, nicotine stimulates the release of dopamine, producing euphoria and creating a sense of pleasure [7]. Also encouraging continued cigarette use is the desire to avoid cigarette withdrawal symptoms, such as irritability, depressed mood, restlessness, anxiety, and somatic symptoms, which can begin as little as 20 min after smoking but generally peak around 2 h [6, 8].

Although smoking prevalence rates are higher for men [2], women* have greater difficulty quitting smoking than men and are less likely to maintain abstinence [9, 10]. One factor that has been hypothesized to contribute to this gender difference relates to women’s exposure to fluctuating reproductive hormones across the menstrual cycle. Indeed, a systematic review and meta-analysis by Weinberger et al. [11] concluded that, relative to the follicular phase, the late luteal phase of the menstrual cycle, which is characterized by a precipitous drop in both estradiol and progesterone, is associated with increased tonic smoking cravings as well as greater withdrawal symptoms during abstinence. However, the studies included in this review were limited by the fact that they did not directly measure ovarian hormone levels; that is, all but one: a study by Schiller and colleagues [12]. In this study, after abstaining from smoking for 12 h, women attended a laboratory session during which they were allowed to smoke ad libitum for 1 h. As they did so, a smoking topography device recorded details about smoking behavior, including number of puffs, mean puff duration, and total puff volume. A blood draw was used to determine serum estradiol and progesterone levels. One to 2 weeks later, participants returned for an identical session and hormone levels were again measured. The results of the study identified a greater decrease in estradiol and progesterone from one measurement point to the other as predictors of more puffs taken and a greater cigarette mass consumed. These results, combined with the conclusions made by Weinberger et al. [11], suggest that withdrawal from estradiol and progesterone may contribute to the enhanced rewarding properties of nicotine seen in the late luteal phase.

While the mechanisms underlying the effect of ovarian hormone withdrawal on the propensity to smoke are not fully understood, one possibility may involve withdrawal from allopregnanolone (ALLO). ALLO is a progesterone-derived neurosteroid that is also positively modulated by estradiol, as estradiol facilitates the conversion of progesterone to ALLO through its effect on several enzymes [13]. ALLO is a positive allosteric modulator of the GABA_A_ receptor [14]; in light of the known tendency for enhanced GABAergic transmission to attenuate dopamine release following nicotine exposure [7], a drop in GABAergic transmission triggered by ALLO withdrawal may underlie the increased propensity to smoke in the late luteal phase.

Thus, it is possible that estradiol and progesterone act together, through ALLO, to increase GABAergic tone and, in turn, attenuate the rewarding properties of nicotine. As the first study to measure ovarian hormone levels in relation to smoking behavior, the study by Schiller et al. [12] represented an important step in clarifying the role that ovarian hormones play in modulating smoking behavior. However, as suggested by Weinberger et al. [11], more research that directly measures ovarian hormone levels is needed. Furthermore, research is needed to determine whether the findings examined in the controlled laboratory setting used by Schiller et al. translate into a detectable relationship between menstrual cycle ovarian hormone changes and smoking in the real world. The current study therefore aimed to prospectively examine the relationship between estrogen and progesterone levels and fluctuation in relation to number of daily cigarettes smoked, as well as women’s self-reported craving for and enjoyment of those cigarettes. To our knowledge, this is the first study to do so. In light of the increased propensity to smoke associated with the late luteal phase, it was predicted that women would report a greater number of cigarettes, more craving, and more enjoyment of cigarettes, following a recent drop in levels of both estradiol and progesterone.

*For the sole purpose of readability, the present paper uses the term "woman" and "female" to refer to an individual with functioning ovaries, including individuals who do not identify as a woman.

## Method

### Participants

Women (*N* = 21) between the ages 18 and 45 reporting regular menstrual cycles lasting 21 to 35 days, reportedly smoking 10 or more cigarettes per day, were recruited. Exclusion criteria included the following: elevated depressive symptoms, defined as a score of 16 or higher on the Center for Epidemiologic Studies Depression Scale (CES-D) [15], use of medications affecting mood or ovarian hormones (e.g., anti-depressants, anxiolytics, hormonal birth control), current use of smoking cessation aids or programs, self-reported psychiatric disorders (e.g., bipolar disorder, psychotic disorder), and pregnancy and lactation (either currently or in the last 12 months). The University of Regina Ethics Board approved this study. Though 22 participants were initially recruited for the study, one participant was excluded due to an excessive amount of missing data (i.e., missing diary submissions and failure to return urine samples) and loss of contact.

### Procedures

#### Enrollment session

During the in-person enrollment session, participants’ eligibility was confirmed and written informed consent was obtained. Participants were provided with ovulation predictor tests, urine collection kits and instructions for both. Relevant demographic information was collected and the Fagerstrom Test for Nicotine Dependence (FTND) [16] was administered. The FTND consists of 6 questions and is a well-established instrument for assessing the intensity of the physical addiction to nicotine. Each answer has a corresponding score which is summed to provide an overall dependency score ranging from low dependence (score of 1–2) to high dependence (score of 8 or higher). The assessment starting point was counter-balanced such that half of the participants began their participation in the study with the follicular phase, while the other half began with ovulation predictor testing and luteal phase testing prior to completing follicular phase testing.

#### Ovulation predictor tests

Starting on cycle day 8, participants began taking daily ovulation predictor tests (Easy@Home Ovulation Test Sticks; Easy@Home, Chicago, IL) until a positive test was obtained, indicating that ovulation would occur in approximately 12–24 h. Therefore, the day following a positive test was considered “ovulation day” and post-ovulation day 1 was considered the first day of the luteal phase. Participants were instructed to send a picture of each test result to the researcher, allowing the researcher to make a final determination on whether the test was positive or not. In the event of an anovulatory cycle, the participant was instructed to continue with ovulation testing until a positive test was obtained. Any luteal phase data already collected was replaced with the data collected after the positive ovulation predictor test.

#### Smoking diary

**Fig. 1:**
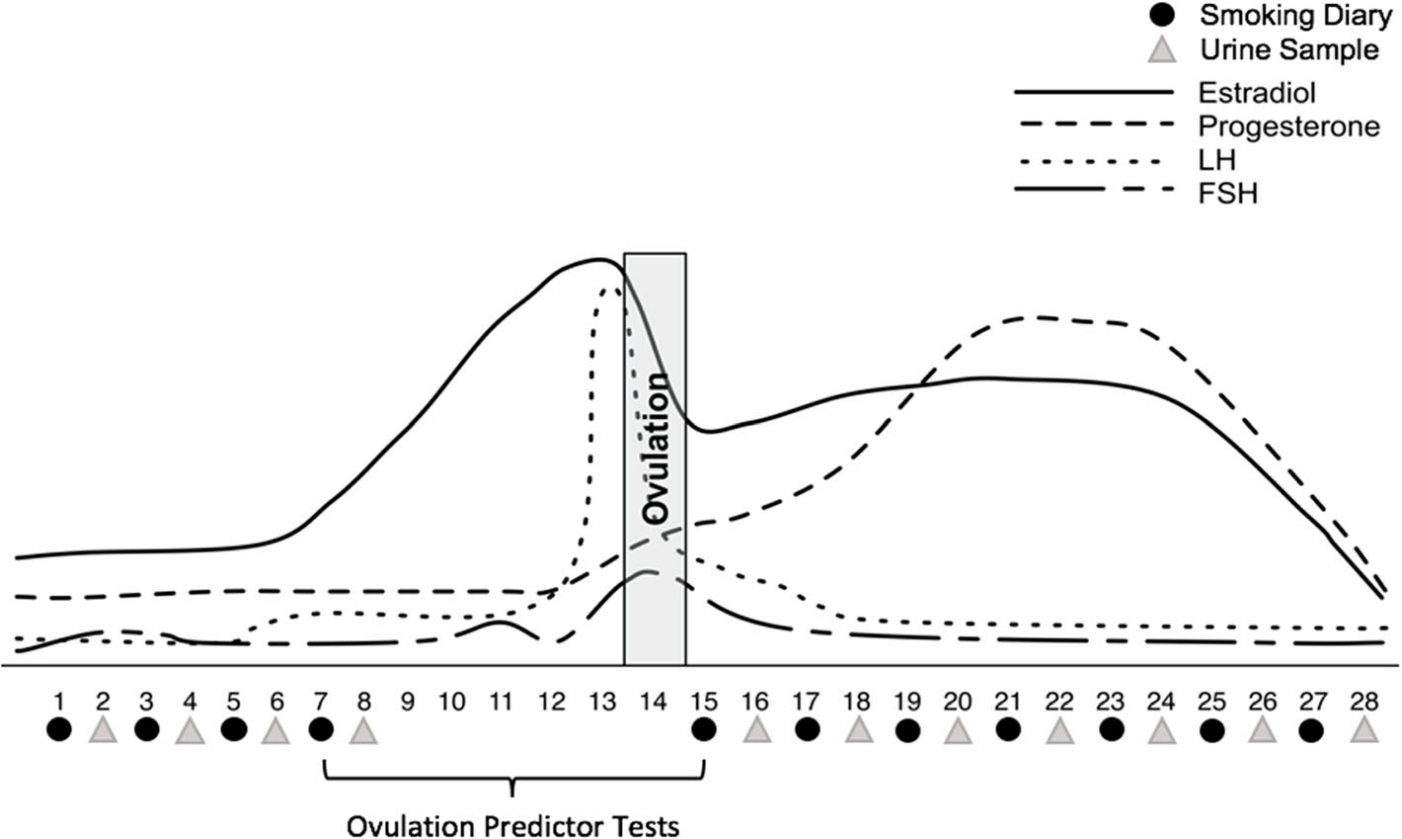
Schematic of the timing of the smoking diary entries and urine sample collections within the menstrual cycle

Smoking behavior was assessed over the course of a menstrual cycle on days 1, 3, 5, and 7 of the follicular phase, in addition to days 1, 3, 5, 7, 9, 11, and 13 post-ovulation (Fig. 1). Specifically, on those days, participants were asked to log into the Expimetrics phone application (Expimetrics Inc., Lafayette, IN) and create a new diary entry each time they smoked a cigarette. The number of total entries served as the number of cigarettes smoked per day. For each entry, participants were also asked to indicate their perceived need (5-point Likert scale ranging from 1—could have done without it to 5—desperate) and their enjoyment (5-point Likert scale ranging from 1—hated it to 5—loved it) of each cigarette. Text message reminders were sent by the researcher on the morning of each tracking day.

#### Urine collection and hormone assays

On the morning following each smoking diary day, participants collected a first-morning voided urine sample, for a total of 11 samples over the course of one menstrual cycle. Samples were stored in the participant’s home freezer until the last urine sample was collected, at which time they were shipped to the lab and stored at −40°C until assayed.

### Hormone assays

Urine samples were assayed for estrone-3-glucuronide (E1G) and pregnanediol glucuronide (PdG), which are the urinary metabolites of estradiol and progesterone, respectively. These metabolites have been shown to correlate very highly (rs = 0.93–0.97 [17]; with serum levels of estradiol and progesterone measured 1 day prior to urine collection. For this reason, urine collection occurred the morning following the smoking diary assessment questionnaire completion.

E1G was assayed using an enzyme immunoassay (Arbor Assays, Ann Arbor, MI), with sensitivity at < 22.5 pg/ml^-1^. The intra-assay and inter-assay variabilities for E1G were 5.1% and 9.1%, respectively. PdG was also assayed using an enzyme immunoassay (Arbor Assays, Ann Arbor, MI), with sensitivity at < 0.180 ng/ml^-1^. The intra-assay and inter-assay variabilities for PDG were 6.5% and 16.0%, respectively.

### Analytic approach

Given the nested data structure of days within participants, Linear Mixed-Effect Modeling (LMEM) was used to analyze the data using a model building approach. The primary outcome variables were (1) the number of cigarettes smoked per day, (2) the perceived need, and (3) perceived enjoyment of each cigarette. Since hormones are well known to follow complex cyclical change patterns [18–20], two different analytic approaches were used to explore the relationship between E1G and PdG with the outcome variables. First, the curvilinear (i.e., quadratic) relationship between current hormone levels and outcome variable was modeled. In this set of models, the hormone levels were person-centered according to each person’s average levels (i.e., each participants’ average hormone level subtracted from each of their daily levels). The second modeling approach sought to capture how daily fluctuations in hormones were related to outcome variables. Change scores were calculated between hormone levels across two successive days, capturing day-to-day variability. For example, positive values reflect an increase that day relative to the previous, while negative values reflect a drop in hormone levels from the previous day. Importantly, both sets of models included an interaction between E1G and PdG, to examine if the influence of one hormone was dependent upon levels of the other. All analyses were conducted using the LMER package in R.

Power calculations were conducted using the methods recommended for repeated measures data [21]. Statistical power in the current study was therefore determined by the sample size, the number of repeated observations, and the intraclass correlation observed between repeated outcome measures. Given 21 participants, 11 repeated measures per participant, and an intraclass correlation coefficient of 0.43 for the daily number of cigarettes smoked, a sample size of 21 allowed for the detection of a medium effect of *f*^2^ = 0.17 where an *f*^2^ of .02 = small effect, .15 = medium effect, and .35 = large effect, as outlined by [22].

## Results

### Participant characteristics

**Fig. 2:**
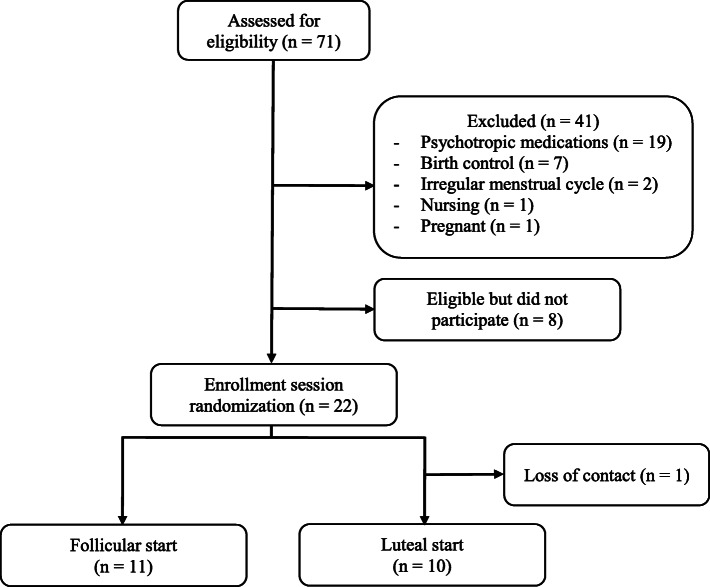
Participant recruitment and counter-balancing randomization

Participant characteristics are summarized in Table 1 and the flow of participants into the study is depicted in Fig. 2. The sample included 21 women who smoked an average of 14 cigarettes daily. All participants had been smoking for at least 1 year, and the average length of time spent smoking was 12 years. A majority of participants were single—never married, white, and working full-time. Most had at least a high school diploma and approximately half had some university education. Participants collected 96% of the required urine samples.

**Table 1:** Demographic and hormonal variables by menstrual phase

Variable		M (SD) or n (%)
Number of cigarettes/day		14.1 (4)
Number of years as an individual who smokes		12.1 (6.7)
Age		29 (7)
Ethnicity	White	18 (86%)
	Aboriginal	2 (9%)
	Other	1 (5%)
Marital status	Single	14 (67%)
	Divorced	1 (5%)
	Married	3 (14%)
	Cohabitating	3 (14%)
Highest level of education	Some high school	3 (14%)
	High school diploma	8 (38%)
	Some university	6 (29%)
	Bachelor’s degree	4 (19%)
Gross household income	No response	4 (19%)
	Less than 19,999	2 (10%)
	20,000 to 34,999	1 (5%)
	35,000 to 49,999	4 (19%)
	50,000 to 69,999	3 (14%)
	70,000 to 112,999	7 (34%)

### Hormone levels by menstrual cycle phase

Hormone levels were compared across four phases: the follicular phase, the early luteal phase (days 1 and 3 post-ovulation), the mid-luteal phase (days 5, 7, and 9 post-ovulation), and the late luteal phase (days 11 and 13 post-ovulation). E1G levels in the follicular phase (M(SE) = 34,961 (7863) pg/ml) were found to be significantly lower than the early luteal (M(SE) = 79,475 (9,750) pg/ml) (p <.001) and mid-luteal phases (M(SE) = 67,213 (8,111) pg/ml) (p <.01). However, they did not significantly differ from levels seen in the late luteal phase (M(SE) = 44,365 (9,767) pg/ml) (p = .389). When examining menstrual cycle differences in PdG, PdG levels in the follicular phase (M(SE) = 1675 (526) pg/ml) were significantly lower than those seen in the mid-luteal phase (M(SE) = 3537 (539) pg/ml) (p = .012) but not significantly different than those seen in the early (M(SE) = 1891(660) pg/ml) (p = .787) or the late luteal phase (M(SE) = 2451(663) pg/ml) (p = .303).

### Hormone change by menstrual cycle phase

As expected, mean E1G change, defined as the change in levels from one assessment point to the next, was positive in the follicular phase (M (SE) = 7574 (5710) pg/ml) as well as the early luteal phase (M(SE) = 588 (9236) pg/ml) and negative in the mid-luteal (M(SE) = −2252 (4820) pg/ml) and late luteal phase (M(SE) = −14,893 (7351) pg/ml). However, only E1G change in the early follicular and late luteal phases were found to be significantly different from one another (p = .022) while the other phases did not differ from each other (ps > .05). The effect of menstrual cycle phase on PdG change did not reach statistical significance (p = .294); however, it did follow expected trends, with PdG change being small in the follicular phase (M(SE) = 21 (439) pg/ml), positive in the early luteal (M(SE) = 331 (698) pg/ml) and mid-luteal (M(SE) = 411 (377) pg/ml) phases and, finally, negative in the late luteal phase (M(SE) = −894 (568) pg/ml).

### The effect of hormone levels on smoking-related outcomes

#### Linear effects

The first set of models explored linear relationships between hormone levels and the outcomes identified above. These models revealed that E1G levels were unrelated to the number of cigarettes smoked but that higher E1G levels were associated with less need for and enjoyment of cigarettes smoked, albeit effects were small (Table 2). PdG levels were unrelated to number of cigarettes, need, or enjoyment (p > .05).

**Table 2:** Linear and quadratic relationships between hormone levels and psychological variables

		Number of cigarettes				Need for cigarettes				Enjoyment of cigarettes		
	Fixed effect β	SE	df	p value	Fixed effect β	SE	df	p value	Fixed effect β	SE	df	p value
*Linear models*												
Intercept	**7.65**	**6.2×10**^**-1**^	**19.9**	**<.001**	−**3.59**	**1.4×10**^**-1**^	**19.87**	**<.001**	**3.44**	**2.1×10**^**-1**^	**19.87**	**<.001**
PdG linear	3.4×10^-5^	5.9×10^-5^	189.2	.559	1.4×10^-5^	1.1×10^-5^	189.1	.201	9.3×10^-6^	1.0×10^-5^	188.9	.361
E1G linear	2.6×10^-6^	4.6×10^-6^	191.2	.580	−**2.5×10**^**-6**^	**8.6×10**^**-7**^	**190.5**	**.005**	−**2.3×10**^**-6**^	**8.0×10**^**-7**^	**189.4**	**.005**
PdG×E1G	−1.3×10^-9^	1.7×10^-9^	197.2	.444	−4.7×10^-10^	3.1×10^-10^	194.8	.133	−4.9×10^-10^	2.9×10^-10^	191.2	.091
*Quadratic models*												
Intercept	**7.53**	**5.9×10**^**-1**^	**19.8**	**<.001**	**3.59**	**1.4×10**^**-1**^	**20.0**	**<.001**	**3.43**	**2.1×10**^**-1**^	**19.9**	**<.001**
PdG linear	−**2.3×10**^**-4**^	**1.1×10**^**-4**^	**190.2**	**.039**	−2.2×10^-5^	2.1×10^-5^	181.1	.310	−2.4×10^-6^	2.0×10^-5^	187.8	.902
PdG quadratic	**1.2×10**^**-8**^	**4.1×10**^**-9**^	**192.4**	**.003**	−3.0×10^-10^	7.8×10^-10^	190.6	.720	4.1×10^-10^	7.3×10^-10^	188.4	.902
E1G linear	−2.8×10^-6^	6.7×10^-6^	191.8	.676	−**2.8×10**^**-6**^	**1.3×10**^**-6**^	**190.3**	**.031**	−**3.2×10**^**-6**^	**1.2×10**^**-6**^	**188.3**	**.007**
E1G quadratic	1.8×10^-11^	2.1×10^-11^	201.6	.391	1.5×10^-12^	4.1×10^-12^	197.3	.713	3.9×10^-12^	3.8×10^-12^	191.5	.304
PdG×E1G	−3.9×10^-9^	2.0×10^-9^	194.3	.052	−3.7×10^-10^	3.8×10^-10^	191.9	.328	−5.1×10^-10^	3.5×10^-10^	189.0	.146

#### Quadratic effects

**Fig. 3:**
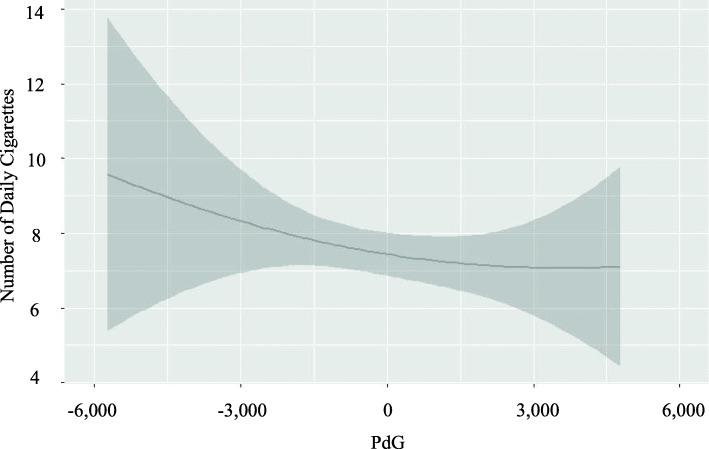
Quadratic model of PdG levels and the number of cigarettes smoked

The inclusion of quadratic terms for the hormone level predictors revealed non-linear relationships between PdG levels and the number of cigarettes smoked. In this model, both the linear and quadratic terms for PdG levels were significant, such that higher levels of PdG predicted lower numbers of cigarettes smoked; however, this effect was only present for below average levels of PdG. That is, when PdG levels were above average, there was no relationship with the number of cigarettes smoked (Fig. 3). Additionally, the interaction term between PdG and E1G was marginally significant such that the negative relationship between each hormone and the number of daily cigarettes smoked was stronger in the presence of higher levels of the other hormone.

Lastly, within the quadratic model, E1G retained its negative linear relationship with both self-reported enjoyment from and self-reported need for cigarettes. The quadratic term was not significant in these models, though its inclusion did increase the effect size of the linear relationships. No other terms in either model were significant. An additional set of models was conducted with random effects on the slope terms to test for between-person variability in the brain-behavior effects. These models did not converge, however, suggesting a lack of between-person variability in these effects.

### The effect of hormone change on smoking-related outcomes

**Fig. 4:**
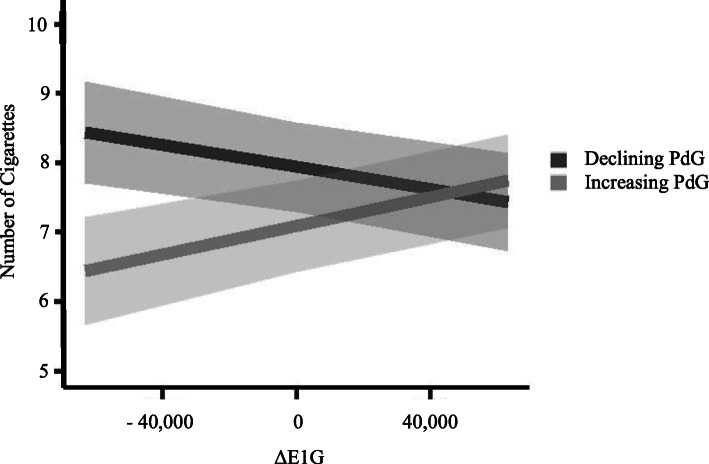
Change score-based statistical modeling of hormone interaction and number of cigarettes smoked

Within the change score model, the change between successive daily readings of hormones was treated as predictors of the outcome variables rather than the actual level from that respective day. This time-series approach isolates the relationship between day-to-day variability in the hormone levels and smoking behavior. This model revealed that while day-to-day fluctuations were not significantly related to any of the three outcomes, there was a significant interaction between E1G change and PdG change on number of cigarettes smoked (Table 3). Further examination of this interaction revealed that the effect of E1G change on number of cigarettes smoked is modified by concurrent PdG change. Specifically, when PdG levels are stable, there is no relationship between changing E1G and the number of cigarettes smoked. However, simultaneously increasing E1G and PdG, as well as simultaneously decreasing E1G and PdG, are associated with an increase in the number of cigarettes smoked (Fig. 4). Thus, the effects of E1G change and PdG change appear to be synergistic. Neither E1G change nor PdG change nor their interaction predicted self-reported need or enjoyment of cigarettes.

**Table 3:** Change score-based statistical modeling of hormone and smoking behavior relationships

		Number of cigarettes				Need for cigarettes				Enjoyment of cigarettes		
	Fixed effect β	SE	df	p value	Fixed effect β	SE	df	p value	Fixed effect β	SE	df	p value
*Intercept*	**7.51**	**6.1×10**^**-1**^	**19.7**	**<.001**	**3.63**	**1.4×10**^**-1**^	**20.04**	**<.001**	**3.43**	**2.1×10**^**-1**^	**19.87**	**<.001**
∆ PdG	−9.8×10^-5^	5.2×10^-5^	179.6	.062	4.4×10^-6^	1.0×10^-5^	179.2	.668	−5.8×10^-6^	9.5×10^-6^	188.9	.543
∆ E1G	1.1×10^-6^	3.3×10^-6^	177.7	.730	−1.0×10^-10^	6.4×10^-7^	177.8	.110	7.8×10^-7^	5.9×10^-7^	189.4	.191
∆ PdG × ∆ E1G	**2.1×10**^**-9**^	**8.7×10**^**-10**^	**189.2**	**.016**	−1.2×10^-10^	1.7×10^-10^	186.4	.486	9.6×10^-12^	1.6×10^-10^	180.8	.952

In addition to examining the relationship between hormone change and smoking levels, the relationship between hormone change and day-to-day change in smoking was also examined. However, no significant effects were obtained.

## Discussion

By their oscillatory nature, hormones are part of an inherently complex biological system, with few studies of their impact on psychological wellness accounting for their cyclical changes in their analytic approaches. This study sought to address this limitation in its exploration of the influences of female reproductive hormones on cigarette smoking behaviors. Indeed, while E1G showed a negative linear relationship with subjective aspects of smoking addiction (the need for and enjoyment of cigarettes), PdG was only related to smoking when non-linear relationships were considered. Specifically, higher PdG levels predicted a lower number of cigarettes smoked; however, this relationship was only found in the context of PdG levels that were below that individual’s average. For days during which PdG levels were above an individual’s average, the relationship between PdG and number of cigarettes smoked was not significant. Concerning the change score models, the relationship between change and number of cigarettes smoked was dependent on PdG change: specifically, a greater number of cigarettes were smoked when E1G and PdG were rising together or falling together. As neither PdG change nor E1G change alone predicted increased smoking behavior, it would appear that the relationship between these two hormones is synergistic, thus highlighting the complexity of the effect of hormones on behavior.

The current findings echo those obtained by Schiller et al. [12] in which a decline in estradiol or progesterone predicted more nicotine consumption. However, there are a few important differences: first, the current study examined the interactions between E1G and PdG and found that simultaneous changes in both hormones translate into greater smoking. Second, our findings suggest that hormonal change in either direction—decreasing or increasing—translates into a greater number of cigarettes smoked. This observation that ovarian hormone change in either direction is associated with greater smoking strongly parallels the known relationship between ovarian hormone change and mood in women, in which case hormone change in either direction is known to trigger depressive mood in certain subsets of women [23]. The most recent research in the area of reproductive psychiatry strongly suggests that fluctuations in ALLO are most likely to mediate the relationship between ovarian hormone change and mood [24, 25]; the same may be true with respect to the propensity to smoke. As mentioned earlier, ALLO is an allosteric modulator of the GABA_A_ receptor. Interestingly, ALLO fluctuation has been shown to result in important changes to the composition of the GABA_A_ receptor subunits, modifying the receptor’s sensitivity. In fact, evidence from the animal literature suggests that ALLO fluctuation can modify the GABA_A_ receptor subunit composition such that ALLO has a paradoxical effect at the GABA_A_ receptor [24]—in other words, ALLO inhibits GABAergic transmission rather than enhances it. In this way, ALLO fluctuation helps to explain how ovarian hormone change in either direction could have the same behavioral effect; it also helps to clarify why tonic levels of ovarian hormones would be independent from the effects of ovarian hormone change. Applied to the current study, it is possible that rapid change in ALLO, driven by changes in estradiol and progesterone, result in a temporary decline in GABAergic tone, which would increase be expected to promote addictive behavior.

The finding of increased smoking in association with a simultaneously decline in E1G and PdG is consistent with the observation that the late luteal phase is accompanied by an increase in smoking behavior while the late follicular phase is not, as the latter is characterized by increasing estradiol but not progesterone [11]. However, it could also suggest that the early luteal post-ovulatory phase may also be associated with increased propensity to smoke. This may help to explain why some studies have found that women tested across the entire luteal phase—not specifically the late luteal phase—exhibit an increased propensity to smoke. While the current study did not detect a significant effect of menstrual cycle phase (follicular and early, mid-, and late luteal) on number of cigarettes smoked, it was likely underpowered to detect such an effect. Larger studies are therefore needed to examine whether smoking increases during this specific phase of the menstrual cycle.

In addition to the finding that simultaneous changes in E1G and PdG increased smoking, a negative linear relationship between E1G and subjective aspects of smoking addiction (the need for and enjoyment from cigarettes) was also observed. This finding is somewhat inconsistent with research in animals suggesting that estrogen has the ability to stimulate dopamine release, thus enhancing the rewarding properties of nicotine [7]. However, animal research has also found that estrogen enhances dopamine release in the striatum and nucleus accumbens in the absence of drug exposure [26]—it is therefore possible that estradiol may attenuate smoking cravings and reward by enhancing basal dopamine transmission. This would be consistent with the findings of one study examining impulsivity across the menstrual cycle and finding that women tend to be less impulsive, a behavioral construct that is highly relevant to the concept of reward-seeking, in the high-estradiol late follicular phase relative to the low-estradiol early follicular phase [27]. A second potential mechanism mediating estradiol’s attenuating effects on smoking cravings may involve an increase in GABAergic transmission via estradiol-induced increases in ALLO [14]. Regardless of the mechanism, though, it is worth noting that estradiol’s effects on cravings were quite small and did not translate into a greater number of cigarettes smoked.

A negative linear relationship between PdG and number of cigarettes smoked was also observed and is largely consistent with prior research suggesting that progesterone reduces the propensity to smoke, likely through its positive effects on ALLO and, in turn, GABAergic transmission (GABA) [25]. However, one new observation made in the current study is that the relationship between progesterone and smoking appears to be stronger in the context of low progesterone levels, perhaps suggesting that the GABA-stimulating effect of increasing progesterone may be more pronounced at low basal progesterone levels.

While these results provide a greater understanding of the influence of ovarian hormones and smoking behavior, they should be considered in light of several limitations. First, the small sample size may limit the generalizability of our findings. It also prevented the examination of between-person differences in the association between smoking and menstrual cycle phases. The potential existence of individual differences in the strength of the relationship between hormones and smoking-related outcomes is worthy of future investigation given the known individual variability in the relationship between ovarian hormones and mood [28]. Second, smoking behavior was reliant on participants' self-report—objective means of assessing smoking behavior such as with the measurement of saliva thiocyanate [29] may have been a more accurate measure of nicotine exposure. Third, the observational nature of this study allows for the possibility that an unmeasured third variable may be driving the relationship between hormonal change and the propensity to smoke; studies that experimentally manipulate hormonal change under controlled conditions would help to more definitively confirm the findings observed here. Despite these limitations, this study has several important strengths. First and foremost, this was the first study to examine the effects of ovarian hormones on the number of cigarettes smoked and on the subjective aspects of smoking behavior. Second, this study employed unique statistical analyses that aimed to capture the oscillatory nature of ovarian hormone fluctuation across the menstrual cycle. Third, the current study counter-balanced the assessment starting point (i.e., follicular versus luteal phase) to ensure that any cycle effects observed were not due to assessment fatigue. Fourth, the current study’s use of ovulation tests was a significant strength as anovulatory cycles can occur even among young women. Finally, despite the aforementioned limitations of the smoking diary, the requirement of having to log every cigarette as it was smoked likely increased the accuracy of recording over past research that had participants log cigarettes retroactively.

### Perspectives and significance

In conclusion, the current study’s findings suggest that simultaneous changes in estradiol and progesterone are associated with an increase in smoking, a finding that may help to explain why the propensity to smoke has been shown to increase in the late luteal phase of the menstrual cycle. Future research should investigate the possibility that smoking propensity also increases in the early luteal post-ovulatory phase given the rise in both estradiol and progesterone that occur during this phase. Further research is also needed to determine the mechanism underlying an effect of ovarian hormone change on smoking propensity. Decreased GABAergic tone resulting from acute changes in ALLO appears to be a worthy candidate of future inquiry.

## Data Availability

The datasets generated and analyzed during the current study are not publicly available due to concerns of patient confidentiality but are available from the corresponding author on reasonable request.
